# Supplementary material for: Regeneration of Osteochondral Defects by Combined Delivery of Synovium-Derived Mesenchymal Stem Cells, TGF-β1 and BMP-4 in Heparin-Conjugated Fibrin Hydrogel
Source: Polymers (Basel). 2022 Dec 7;14(24):5343. doi: 10.3390/polym14245343 (PMC9780905; doi:10.3390/polym14245343)
Supplement: Supplementary file 1 [file polymers-14-05343-s001.zip › polymers-1855253-supplementary.pdf]

**Supplementary materials:**

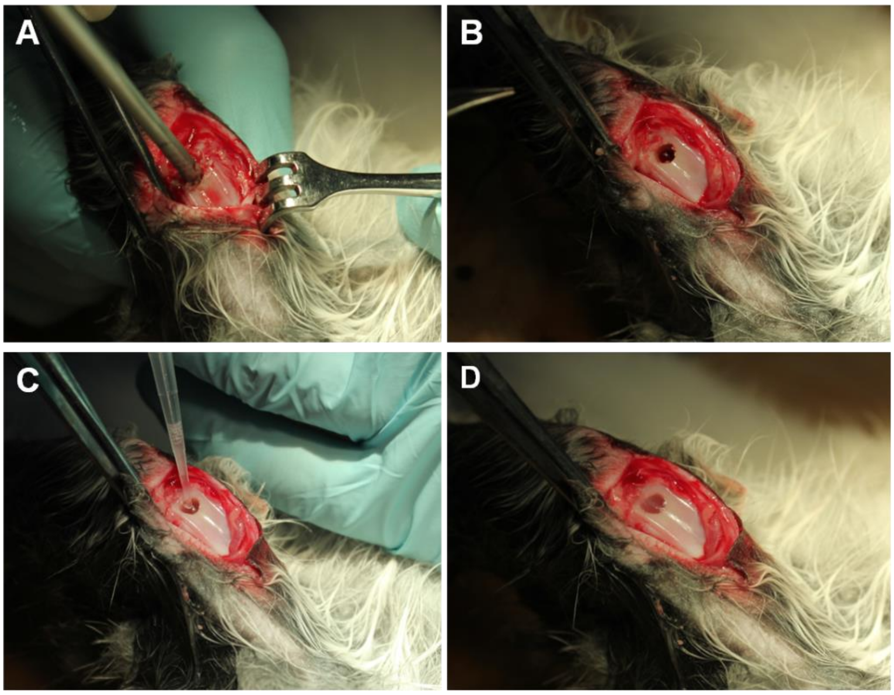


**Figure S1.** The surgery procedure stages for the establishment of osteochondral defect and HCF hydrogel implantation. (**A, B**) Osteochondral defect creation in a patello-femoral bone in the patella groove of a rabbit knee joint with a handle-operated drill. (**C**) Implantation of hydrogel into the osteochondral defect. (**D**) Implanted HCF hydrogel after gelation in the osteochondral defect.

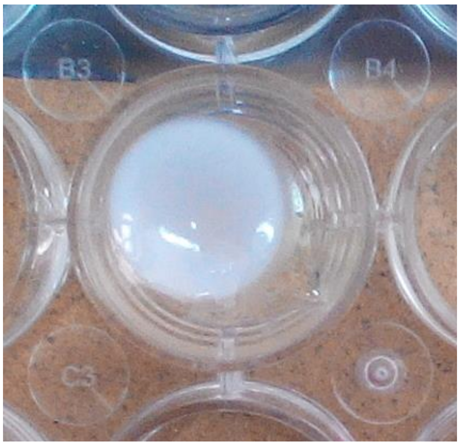


**Figure S2.** Representative image of gross appearance of HCF hydrogel after gelation

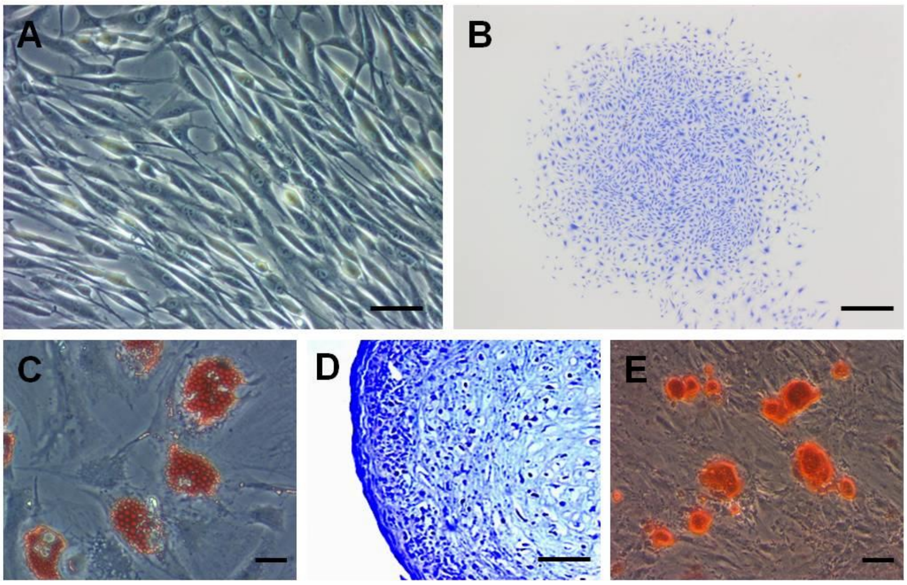


**Figure S3.** Characterization of rabbit SDMSCs. (**A**) Morphology of primary culture of rabbit SDMSCs. Phase-contrast image. Scale bar, 20 μm. (**B**) *Colony forming* ability of SDMSCs. Colonies were stained with crystal violet. Scale bar, 200 μm. (**C**) Adipogenic differentiation of SDMSCs. Adipocytes were stained with Oil Red O. Scale bar, 20 μm. (**D**) Chondrogenic differentiation of SDMSCs. Cross section of SDMSC pellet was stained with Toluidine blue. Scale bar, 50 μm. (**E**) Osteogenic differentiation of SDMSCs. Osteoblasts were stained with Alizarin Red S. Scale bar, 20 μm.
